# Integrating treatment cost reduction strategies and biomarker research to reduce costs and personalize expensive treatments: an example of a self-funding trial in non-small cell lung cancer

**DOI:** 10.3389/fphar.2023.1274532

**Published:** 2023-11-28

**Authors:** Alessandra I. G. Buma, Berber Piet, Rob ter Heine, Michel M. van den Heuvel

**Affiliations:** ^1^ Radboud University Medical Centre, Department of Respiratory Medicine, Nijmegen, Netherlands; ^2^ Radboud University Medical Centre, Department of Pharmacy, Nijmegen, Netherlands

**Keywords:** personalized treatment, expensive treatment, treatment cost reduction strategies, sustainable healthcare, biomarker research

## Abstract

Personalization of treatment offers the opportunity to treat patients more effectively based on their dominant disease-specific features. The increasing number and types of treatment, and the high costs associated with these treatments, however, demand new approaches that improve patient selection while reducing treatment-associated costs to ensure sustainable healthcare. The DEDICATION-1 trial has been designed to investigate the non-inferiority of lower dosing regimens when compared to standard of care dosing regimens as a potential effective treatment cost reduction strategy to reduce costs of treatment with expensive immune checkpoint inhibitors in non-small cell lung cancer. If non-inferiority is confirmed, lower dosing regimens could be implemented for all therapeutic indications of pembrolizumab. The cost savings obtained within the trial are partly reinvested in biomarker research to improve the personalization of pembrolizumab treatment. The implementation of these biomarkers will potentially lead to additional cost savings by preventing ineffective pembrolizumab exposure, thereby further reducing the financial pressure on healthcare systems. The concepts discussed within this perspective can be applied both to other anticancer agents, as well as to treatments prescribed outside the oncology field.

## 1 Introduction

The accumulated body of research and large number of new available treatment options have allowed for a personalization of treatment within multiple therapeutic areas (Zugazagoitia et al., 2016; Schee Genannt Halfmann et al., 2017; Yamamoto et al., 2022). This way, patients can be treated more effectively at the individual patient level based on their dominant disease-specific features (Mathur and Sutton, 2017). Other advantages comprise minimization of overtreatment, avoidance of adverse events, prevention of a delay in administering alternative treatment options, and a potentially marked reduction in overall treatment-associated costs by preventing the administration of ineffective treatment to specific patient subgroups (Jakka and Rossbach, 2013; Cherny et al., 2014; Morkovich, 2023). Two major issues, however, comprise (a) the still limited understanding of the complex underlying biological pathways involved in many diseases, thereby complicating an accurate upfront or early identification of responders to specific treatments, and (b) the high costs of most new treatments (Jakka and Rossbach, 2013; Goetz and Schork, 2018). As a consequence, the sustainability of healthcare systems is increasingly threatened (Jakka and Rossbach, 2013; Goetz and Schork, 2018). New approaches that help improving patient selection while reducing treatment-associated costs in clinical practice are urgently needed (Jakka and Rossbach, 2013; van Ommen-Nijhof et al., 2021; Superchi et al., 2022).

Biomarkers are considered to be essential for the personalization of treatment since they can be used as indicators of pathophysiological processes or pharmacological responses to a therapeutic intervention (Sarhadi and Armengol, 2022; Morkovich, 2023). Their characteristics make them useful in “providing the right treatment to the right patient, at the right dose, at the right time”, thereby preventing unnecessary exposure in patients who do not benefit from a specific treatment. Simultaneously, biomarkers can help obtain valuable insights into the pathophysiological mechanisms underlying the disease of interest (Mishra and Verma, 2010; Landeck et al., 2016). In this perspective, we present an example of a novel, self-funding trial design that integrates both treatment cost reduction strategies and biomarker research to reduce costs and improve personalization of treatment with expensive immune checkpoint inhibitors (ICIs) in advanced non-small cell lung cancer (NSCLC). The concepts discussed within this perspective can be applied both to other anticancer agents, as well as to treatments prescribed outside the oncology field.

## 2 Overview of the DEDICATION-1 (NVALT 30) trial

**FIGURE 1:**
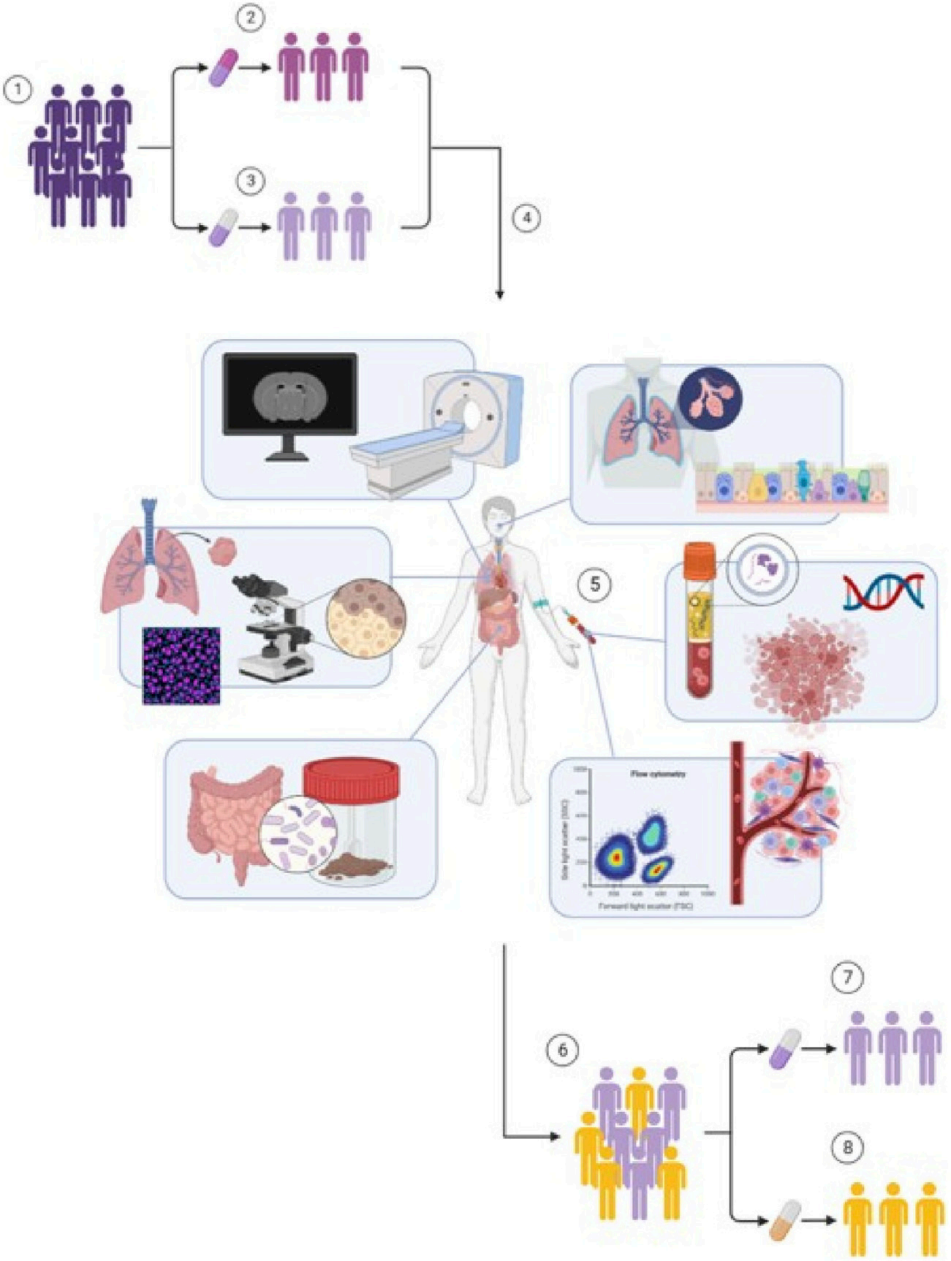
Design of the DEDICATION-1 trial. (1) Advanced NSCLC patients without targetable driver mutations eligible for pembrolizumab-containing treatment are randomized in a 1:1 ratio between (2) standard of care regimens and (3) lower dosing regimens of pembrolizumab treatment. (4) Simultaneously, all patients are also included in the biomarker sub-study embedded within the DEDICATION-1 trial. (5) Within this biomarker sub-study, extensive biomarker research is performed that investigates the utility of liquid biopsies, proteomics, pharmacokinetics and immunopharmacology, exhaled breath, AI-based lung imaging, computational pathology, and the microbiome, in predicting (non-)response to pembrolizumab-containing treatment. (6) The implementation of these biomarkers will result in an accurate identification of (7) responders–who will be treated with lower dosing regimens of pembrolizumab treatment if non-inferiority is confirmed–and (8) non-responders–who can receive alternative and possibly more effective treatments –, thereby improving personalization of pembrolizumab-containing treatment and further reducing pembrolizumab exposure and treatment costs in non-responding patients. Abbreviations: NSCLC, non-small cell lung cancer; DEDICATION-1, Dose tapering and Early Discontinuation to InCreAse cosT-effectIveness Of immunotherapy for NSCLC; AI, artificial intelligence.

In advanced NSCLC patients without targetable driver mutations, different ICIs have been approved and introduced in clinical practice (Twomey and Zhang, 2021). We designed a nationwide multi-center open label randomized non-inferiority trial named “Dose tapering and Early Discontinuation to InCreAse cosT-effectIveness Of immunotherapy for NSCLC” (DEDICATION-1) (NCT04909684) that includes advanced NSCLC patients who are eligible for first-line pembrolizumab-containing treatment in the Netherlands (Figure 1). Pembrolizumab is a fully humanized immunoglobulin G4 monoclonal antibody that is directed against the programmed death-1 (PD-1) receptor, preventing its interaction with programmed death-ligand 1 (PD-L1) and PD-L2, thereby increasing the antitumor immune response (Renner et al., 2019). Based on their tumour PD-L1 expression, patients receive either pembrolizumab monotherapy (PD-L1 expression ≥50%) or pembrolizumab in combination with platinum-based doublet chemotherapy (PD-L1 expression <50%) (Reck et al., 2019; Gadgeel et al., 2020). The primary aim of the trial is to investigate the non-inferiority of a reduced dose *versus* the standard of care dose of pembrolizumab for treatment of advanced stage NSCLC in terms of 1-year overall survival (OS). The secondary aim includes the development of biomarkers predicting (non-)response to pembrolizumab-containing treatment. Currently, 25–30 Dutch sites–both academic and non-academic–are participating in the trial. The following sections will elaborate on the rationale and design of the trial, and the parties involved in the trial.

### 2.1 Dosing rationale of the DEDICATION-1 (NVALT 30) trial

Dose and schedule selection for ICIs has shown to be challenging since there is no clear dose-response relationship, the toxicity profile of ICIs markedly differs from that of cytotoxic agents, and exposure-toxicity relationships are not yet well understood (Agrawal et al., 2016). Pembrolizumab treatment was initially approved by the US Food and Drug Administration (FDA) in a weight-based dosing schedule of 2 mg/kg every 3 weeks (Q3W) based on results obtained in a phase I trial that investigated pembrolizumab doses up to 10 mg/kg every 2 weeks (Q2W) (Jiang et al., 2022). The trial showed complete peripheral PD-1 target engagement at doses of 1 mg/kg or higher–confirmed by an *ex-vivo* interleukin-2 (IL-2) stimulation test–and no differences in durable anti-tumour activity and dose-limiting toxicities were seen at doses from 1 to 10 mg/kg Q2W (Renner et al., 2019; Low et al., 2021; Hirsch et al., 2022). In addition, no differences in response rates between doses of 2 mg/kg Q3W and higher were observed in the subsequent expansion cohorts, implying that increasing pembrolizumab dose from 2 mg/kg to higher does not contribute to tumour control (Low et al., 2021; Hirsch et al., 2022). Since doses lower than 2 mg/kg were not examined, it remains unknown whether systemic exposure associated with doses lower than 2 mg/kg Q3W results in sufficient intratumoral PD-1 inhibition and, therefore, in effective treatment (Li et al., 2021; Low et al., 2021).

To enhance convenience and reduce spill of partially used vials, pembrolizumab treatment was later also approved in a fixed dosing schedule of 200 mg Q3W or a high-dose, extended-interval dosing schedule of 400 mg every 6 weeks (Q6W) based on results obtained in *in silico* investigations (Freshwater et al., 2017; Jiang et al., 2022). Note that these investigations showed that a fixed dose of 150 mg Q3W–and not 200 mg Q3W–resulted in pharmacokinetically equivalent exposure as the initially approved dose of 2 mg/kg Q3W (Freshwater et al., 2017). With ever increasing restrictions on healthcare budgets and the high costs associated with pembrolizumab treatment, a re-evaluation of the current dosing regimens has often been suggested (Jiang et al., 2022).

**FIGURE 2:**
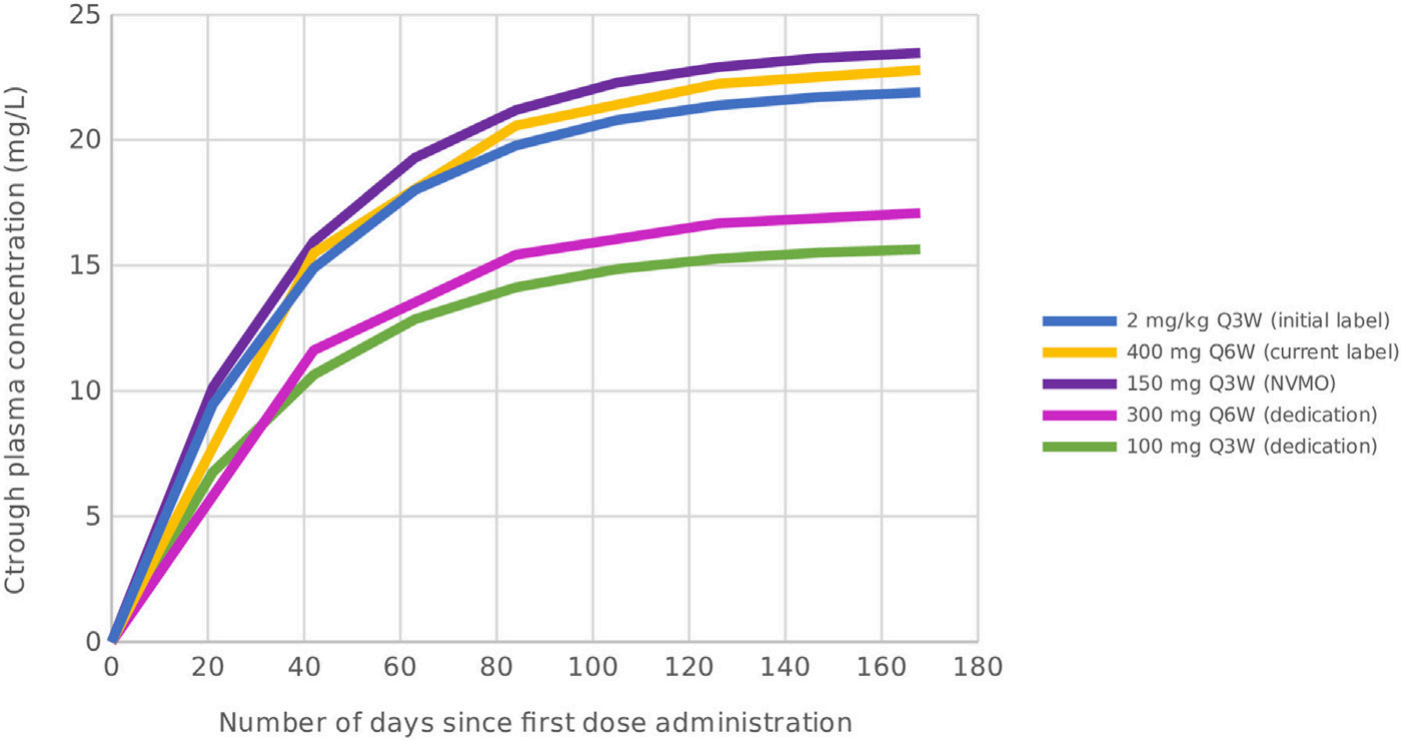
Simulated C_trough_ levels of pembrolizumab for the initially approved dosing regimen, and the standard of care and lower dosing regimens investigated within the DEDICATION-1 trial. Based on the simulated *C*_*trough*_ levels, the 150 mg Q3W and 100 mg Q3W dosing regimens can be considered pharmacokinetically equivalent to the 400 mg Q6W and 300 mg Q3W dosing regimens, respectively. Abbreviations: C_trough_, trough plasma concentration; Q3W, every 3 weeks; Q6W, every 6 weeks; NVMO, Nederlandse Vereniging voor Medische Oncologie; DEDICATION-1, Dose tapering and Early Discontinuation to InCreAse cosT-effectIveness Of immunotherapy for NSCLC.

The DEDICATION-1 (NVALT 30) trial has been designed to investigate whether treatment with lower dosing regimens is non-inferior to treatment with standard of care dosing regimens. Advanced NSCLC patients eligible for pembrolizumab-containing treatment are randomized in a 1:1 ratio between standard of care and lower dosing regimens of pembrolizumab treatment (Figure 1). The standard of care dosing regimens comprise the currently registered 400 mg Q6W dosing regimen and a 150 mg Q3W dosing regimen. The lower dosing regimens consist of a 300 mg Q6W and a 100 mg Q3W dosing regimen. Note that the 150 mg Q3W and 100 mg Q3W dosing regimens can be considered pharmacokinetically equivalent to the 400 mg Q6W and 300 mg Q3W dosing regimens, respectively, based on simulated trough plasma concentration (C_trough_) levels (Figure 2). Since PD-1 inhibition directly correlates with pembrolizumab concentration and the concentration level is lowest just before the next administered dose, it is hypothesized that the C_trough_ level is the most informative pharmacological parameter to predict treatment efficacy (Li et al., 2021). Hence, no difference in efficacy is expected between the pharmacokinetically equivalent dosing regimens investigated within the trial.

Pembrolizumab is currently commercially available in 4 mL vials, corresponding to a 100 mg dose per vial (each ml of concentrate contains 25 mg of pembrolizumab) (European Medicines Agency, 2015). This would result in only partially used vials for each patient if the lower dosing regimen of 300 mg Q6W would be applied. In 2020, however, employees of Merck published an article on the physiochemical stability of pembrolizumab admixture solution (25). Results showed that pembrolizumab has a longer shelf-life than currently stated in the package leaflet, if adequate aseptic conditions can be maintained during reconstitution (Sundaramurthi et al., 2020). This enables the use of a single vial for multiple patients, thereby preventing unnecessary costs due to spill of only partially used vials when applying the lower dosing regimens.

### 2.2 Design and sample size calculation of the DEDICATION-1 (NVALT 30) trial

According to the US FDA guidance on pharmacokinetic-based criteria for supporting alternative dosing regimens of PD-1 and PD-L1 inhibitors, lower dosing regimens cannot be considered pharmacokinetically equivalent to the standard of care dosing regimens if they are expected to result in more than 20% lower exposure (U.S. Department of Health and Human Services, 2022). Additional clinical data to support efficacy of the proposed lower dosing regimens are then considered necessary (U.S. Department of Health and Human Services, 2022). In line with the practical recommendations on the level of evidence needed to apply alternative dosing regimens in clinical practice published by *Overbeek et al.*, we selected a prospective non-inferiority design to provide high quality evidence for lower dosing of pembrolizumab treatment (Overbeek et al., 2023).

Based on the European Medicines Agency (EMA) guidelines for performing non-inferiority trials, the lower dosing regimens can only be defined non-inferior to the standard of care dosing regimens if the following two criteria are simultaneously met: (a) The efficacy of the lower dosing regimens is allowed to be worse than the standard of care dosing regimens if the difference is within a pre-specified clinically relevant boundary, and (b) the lower dosing regimens must still be superior to the treatment used as control in the trials that led to registration of the standard of care dosing regimens (Committee for medical products for human use CHMP, 2005). In our trial, non-inferiority is confirmed if (a) with 95% one-sided confidence the absolute difference in 1-year OS rate is below 10%, and (b) with 95% two-sided confidence the 1-year OS in the lower dosing regimens arm is superior to that of a virtual cohort of patients receiving chemotherapy. The 1-year OS rate in the virtual cohort of patients receiving chemotherapy will be estimated based on the KEYNOTE-024 and KEYNOTE-189 studies with a ratio between patients with a tumour PD-L1 expression <50% and ≥50% equal to that observed in our trial (Committee for medical products for human use CHMP, 2005).

Based on the abovementioned criteria, the inclusion of 750 patients who are followed for at least 1 year is needed to yield (a) 90% power to declare non-inferiority according to the first criterion–assuming an equal true 1-year OS rate on both treatment regimens of 70%–and (b) 91% power to find non-inferiority when the percentage of patients with a tumour PD-L1 expression ≥50% is lower than 75% according to the second criterion. The power of the trial will drop in case the percentage of patients with a tumour PD-L1 expression ≥50% is higher than the estimated 75%, or if the true 1-year OS rate is lower than the estimated 70%. The worst case–still assuming an equal 1-year OS rate in both arms–would appear if the true survival rate is 50%. This would yield a power of 86% to declare non-inferiority.

An interim analysis will be performed after the first 250 patients have been included and followed for at least 1 year. Inclusion in the trial will be stopped early if among these patients a difference of 10% or higher in 1-year OS rate is observed in favour of the standard of care dosing regimens. Patients already included in the trial at that time point will still be followed until 1 year after inclusion for the final analysis. The stopping boundary of 10% corresponds to a conditional power of 5%. This is relatively low when compared to the conditional power of 15%–which corresponds to a stopping boundary of 8%–usually applied for futility analyses. However, we considered a stopping boundary of at least 10% to be clinically relevant. An early stopping rule for efficacy is not considered to be necessary since we do not expect the lower dosing regimens to be superior to the standard of care dosing regimens.

### 2.3 Biomarker research and development within the DEDICATION-1 (NVALT 30) trial

Pembrolizumab-containing treatment is currently prescribed as a non-personalized first-line treatment, since it has been approved for all advanced NSCLC patients who lack targetable driver mutations regardless of their tumour PD-L1 expression (Reck et al., 2019; Gadgeel et al., 2020). In clinical practice, however, only half of these patients experience a clinical benefit (Grizzi et al., 2017; Reck et al., 2019). As a result, a large proportion of patients is unnecessarily exposed to potential treatment-related adverse events, and will not receive alternative–and potentially more effective–treatment options for this rapidly progressing disease (Cherny et al., 2014; Morkovich, 2023). The cost savings obtained by investigating pembrolizumab dose reduction are, therefore, not only being used to fund the clinical trial itself, but also to fund the biomarker sub-study that is embedded within the DEDICATION-1 trial to improve personalization of pembrolizumab treatment through accurate patient selection (Figure 1).

Due to the complexity of the mechanism of action of ICIs and the many factors that influence a patient’s likelihood to response, it is expected that more than one biomarker will be needed to improve patient selection and clinical decision making (Blank et al., 2016). Therefore, the DEDICATION-1 trial has been designed to serve as a platform for extensive biomarker research that investigates multiple biomarkers (e.g., liquid biopsies, proteomics, pharmacokinetics and immunopharmacology, exhaled breath, artificial intelligence (AI)-based lung imaging, computational pathology, and the microbiome) in order to assess their utility–both individually and within the context of a compound biomarker–in predicting (non-)response to pembrolizumab-containing treatment. Importantly, the trial design allows for the development of predictive biomarkers that are able to identify both primary treatment resistance (e.g., predictive biomarkers that predict (non-)response before start or early after start of treatment) and secondary treatment resistance (e.g., monitoring biomarkers that can be applied to identify (non-)response during course of treatment) (Buma et al., 2021; van Delft et al., 2022; Buma et al., 2023). In parallel, an early Health Technology Assessment (HTA) analysis is being performed to assess the value of biomarker-guided treatment selection by providing high-quality research information on the effectiveness, costs, and impact of the implementation of such an approach (Ferraro et al., 2022). This way, the investigated biomarkers not only provide valuable new insights on the pathophysiological mechanisms underlying advanced NSCLC disease and the pharmacological behaviour of ICI agents, but simultaneously have a high chance of being actually implemented in clinical practice to guide appropriate prescription of pembrolizumab-containing treatment, and facilitate patient education and counseling.

## 3 Self-funding–a double edged sword to improve sustainable healthcare by public parties

In current practice, new drugs are being developed by pharmaceutical companies alongside with companion diagnostics if available. As soon as the drug has entered the market, the need for further optimization and personalization of treatment is often hampered by the commercial interests of these companies. There is no intrinsic motivation other than increasing or continuing their market share. However, healthcare providers and other public parties, responsible for creating an affordable and sustainable healthcare system, do feel the motivation to further fine tune the treatment.

The DEDICATION-1 trial is a unique joint-venture of public parties who pursue affordable and sustainable healthcare. The parties include (a) healthcare professionals, who are directly involved in clinical care or management of patients, (b) healthcare insurers, who are essential in providing access to the alternative dosing regimens, and (c) the patient advocate organization Longkanker Nederland, who meets the needs of the lung cancer patients for which the alternative dosing regimen has been proposed. The trial is additionally supported by the Dutch healthcare professional associations Nederlandse Vereniging van Artsen voor Longziekten en Tuberculose (NVALT) and Nederlandse Vereniging van ZiekenhuisApothekers (NVZA). Collaboration between these public parties and national healthcare associations is crucial to structurally perform trials like the DEDICATION-1 and to increase adherence if cost-effective dosing regimens are implemented in clinical practice. External funding of the trial is provided by the Treatmeds foundation, which is an initiative of the Dutch healthcare insurers and aims to keep expensive treatments affordable and available, by financially supporting approaches that reduce high treatment costs while maintaining treatment efficacy.

## 4 Discussion

The increasing number and types of available treatment options, and the high costs of these new treatments, demand new approaches that improve patient selection while reducing treatment-associated costs to ensure sustainable healthcare (Jakka and Rossbach, 2013; van Ommen-Nijhof et al., 2021; Superchi et al., 2022; van Till et al., 2022). Within the DEDICATION-1 trial, we apply lower dosing of pembrolizumab as a potential effective strategy to reduce pembrolizumab treatment-associated costs. The cost savings are partly reinvested in biomarker research in order to improve the personalization of treatment through an upfront or early identification of patients who might benefit from it. The implementation of these biomarkers will potentially lead to additional cost reductions due to prevention of ineffective pembrolizumab exposure, thereby further reducing the financial pressure on healthcare systems.

Pembrolizumab is currently prescribed for many different solid malignancies (Stewart, 2021). Based on data obtained in nivolumab, which also targets PD-1, one could argue that higher doses of anti-PD-1 treatment are required to achieve optimal efficacy in NSCLC when compared to other malignancies (Agrawal et al., 2016). This would imply that lower dosing regimens could also be implemented for all therapeutic indications without compromising efficacy if non-inferiority in NSCLC is confirmed (Renner et al., 2019; Jiang et al., 2022). This would substantially decrease the significant costs associated with global pembrolizumab prescription. On the other hand, we expect that biomarkers do vary for the different therapeutic indications. Each cancer type is characterized by unique molecular and histopathological features (Hoadley et al., 2014; Komura et al., 2022). This may result in distinct features associated with (non-)response to pembrolizumab-containing treatment, thus requiring different (combinations of) predictive or monitoring biomarkers. For instance, a different set of serum tumour markers is valuable for monitoring treatment response in NSCLC when compared to breast or colorectal cancer (Duffy, 2006; Jelski and Mroczko, 2020). Consequently, the application of a prediction model developed for identifying (non-)response in NSCLC will need to be adapted for other cancer types. The cost savings obtained through the universal application of lower pembrolizumab dosing could be used to develop cancer type-specific biomarkers that improve personalization of pembrolizumab-containing treatment in cancers other than NSCLC.

The integration of treatment cost reduction strategies and biomarker research can also be applied to improve personalization of other treatments even outside the oncology field. Different strategies have already shown to be effective for cost reduction of several anticancer agents (Serritella et al., 2020). Abiraterone, for example, is an enzyme inhibitor indicated for prostate cancer which has a large food effect (Ratain, 2011). Results obtained within a randomized non-inferiority trial showed abiraterone administration at 250 mg with a low-fat meal to be non-inferior in clinical endpoints and pharmacodynamic effects when compared to standard of care administration at 1,000 mg while fasting (Szmulewitz et al., 2018). Another example is the application of shorter adjuvant treatment duration in breast cancer patients who can be treated with six instead of 12 months of adjuvant trastuzumab, and in colon cancer patients in whom 3 months of adjuvant chemotherapy was shown to be as effective as 6 months (Grothey et al., 2018; Earl et al., 2019). Note that in the current era of personalized medicine, the drugs in these examples–and most of other currently available treatments–are still prescribed applying a one-size-fits-all approach as for pembrolizumab-containing treatment. Cost reduction strategies could therefore not only be used to reduce financial pressure on healthcare systems, but also to improve the personalization of a high number of treatments by funding the development and implementation of companion biomarkers that guide treatment selection and therapeutic monitoring in clinical practice. In addition, the increased knowledge gained on the pathophysiological mechanisms underlying the disease of interest could possibly help develop new and more effective treatment options.

The DEDICATION-1 trial is also an example of a framework that can be adopted to effectively reduce current treatment costs and improve personalization of treatments in the short-term. However, one could argue that the concept of the DEDICATION-1 trial is simply a direct consequence of our current healthcare price setting and regulation system. Whether the implementation of this framework will thus be effective on the long-term, is unknown. Until sustainable solutions for drug pricing and healthcare reimbursement are implemented, trials like the DEDICATION-1 can be performed to develop lower-cost and personalized treatment regimens (Uyl-de Groot and Löwenberg, 2018).

In conclusion, we presented the DEDICATION-1 trial as an example of a novel, self-funding trial design that integrates both treatment cost reduction strategies and biomarker research to reduce costs and improve personalization of treatment with expensive ICIs in advanced non-small cell lung cancer (NSCLC). The concepts discussed within this perspective can be applied both to other anticancer agents, as well as to treatments prescribed in other therapeutic areas in order to improve their personalization and cost-effectiveness in the short-term.

## The DEDICATION-1 Consortium

Paul Brinkman, Anke H. Maitland-van der Zee (Department of Respiratory Medicine, Amsterdam University Medical Centre); Daan van den Broek, Huub van Rossum (Department of Laboratory Medicine, Netherlands Cancer Institute); Sjaak Burgers (Department of Respiratory Medicine, Netherlands Cancer Institute); Francesco Ciompi, Katrien Grünberg (Department of Pathology, Radboud University Medical Center); Simona M. Cristescu (Institute for Molecules and Materials, Radboud University); Bram van Ginneken, Colin Jacobs (Diagnostic Image Analysis Group, Radboud University Medical Center); Lizza Hendriks (Department of Respiratory Medicine, Maastricht University Medical Center); Jeroen Hiltermann (Department of Respiratory Medicine, University Medical Center Groningen); Firdaus Mohamed Housein (Department of Radiology, University Medical Center Utrecht); Alwin Huitema (Department of Pharmacy and Pharmacology, Netherlands Cancer Institute); Jakko van Inge (Department of Medical Microbiology, Radboud University Medical Center); Hans Koenen, Ruben Smeets (Department of Laboratory Medicine, Radboud University Medical Center); Marjolijn Ligtenberg (Department of Tumor Genetics, Radboud University Medical Center); Vincent van der Noort (Department of Statistics, Netherlands Cancer Institute); Mathias Prokop (Department of Radiology and Nuclear Medicine, Radboud University Medical Center); Heinrich Roder (Biodesix); Valesca Rètel (Department of Psychosocial Research and Epidemiology, Netherlands Cancer Institute); Thomas Würdinger (Department of Neurosurgery, Amsterdam University Medical Center and Free University Medical Center).

## Data Availability

The original contributions presented in the study are included in the article, further inquiries can be directed to the corresponding author.
